# Different experiences of weight management and physical activity during pregnancy - a qualitative study of women and healthcare professionals in Australia

**DOI:** 10.1080/17482631.2023.2202973

**Published:** 2023-04-26

**Authors:** Catherine Knight-Agarwal, Michelle Minehan, Bridget Cockburn, Sophie Cashel, Monica Yuri Takito

**Affiliations:** a Department of Nutrition and Dietetics, The University of Canberra, Canberra, Australia; b Department of Human Movement, School of Physical Education and Sport, The University of São Paulo, Sao Paulo, Brazil

**Keywords:** Antenatal Care, Pregnancy, Exercise, Weight management, Lifestyle, Health Knowledge, Attitudes, Practice

## Abstract

Objective: Pregnancy is often described as a pivotal life stage for women, where regular contact with health professionals may play an important role in lifestyle awareness. This study explored the knowledge, practices, and beliefs of health professionals and pregnant women regarding physical activity and weight management during the antenatal period. Methods: A qualitative study was undertaken in southeastern Australia using individual interviews. Recruitment sought women of gestation >12 weeks, experiencing an uncomplicated pregnancy (*n* = 6), and antenatal health professionals including midwives (*n* = 4) and an obstetrician (*n* = 1). Data were analysed using Interpretive Phenomenological Analysis. Results: Three major themes emerged: (1) women rely on multiple sources of pregnancy-related healthy lifestyle information; (2) discussions around healthy lifestyle behaviours are low priority and often inconsistent; and (3) lifestyle-related topics perceived as sensitive make some conversations and actions difficult. Conclusions: Pregnant women expressed gaps in lifestyle-related knowledge and education being provided by health professionals. In turn, health professionals expressed difficulty discussing sensitive topics such as weight with pregnant women and had limited knowledge of pregnancy-specific physical activity guidelines. The themes generated by this study may form the foundation for further research to inform clinical policy and practice regarding advice in antenatal care.

## 1. Introduction

Pregnancy is a unique period in a woman’s life. A time where she may be more motivated than usual to undertake healthy lifestyle activities. In 2014, more than 2.5 million Australian women of childbearing age (≥18 years) were overweight (approximately 29%) (defined as a body mass index [BMI] of 25–29.9 kg/m^2^), and close to 2.5 million women were classified as obese (approximately 27%) (BMI of ≥ 30 kg/m^2^) (Australian Bureau of Statistics, 2015; Australian Institute of Health and Welfare, 2017). Of concern is that at least one-third of Australian pregnant women are overweight at the time of conception, with 46% having given birth in 2014 being overweight or obese (De Jersey et al., 2013). Excess energy consumption in tandem with lower than recommended levels of energy expenditure places women at high risk of unhealthy gestational weight gain (GWG) (Bookari et al., 2016b). Maternal obesity and excess GWG have been associated with poor health outcomes for both mother and child, including gestational diabetes mellitus (GDM), perinatal depression, low rates of breast-feeding, miscarriage, hypertensive thromboembolic disorders, birthing complications (resulting in caesarean sections), still birth, macrosomia, and increased risk of offspring developing chronic disease later in life (Arrish et al., 2016).

Considering the array of potential adverse pregnancy outcomes associated with overweight and obesity, it is important to learn where women source health information from. Understanding such sources can provide researchers with opportunities to ensure women have access to evidence-based guidance. Books, personal experience, family, friends, and prenatal classes have all been reported to contribute to women’s knowledge regarding weight management during pregnancy (Arrish et al., 2017a; Kraschnewski et al., 2014). It is well documented that a healthy diet plus regular physical activity is equally important for appropriate gestational weight management. Nevertheless, a recent review examined pregnancy-related nutrition and physical activity resources available on Australian government and leading industry body websites. The authors reported that websites containing nutritional information were more readily found than those providing information on physical activity (Cannon et al., 2020).

In addition, an Australian study investigating GWG found that pregnant women possessed limited knowledge of credible weight management recommendations, suggesting a need to address this issue through health-care providers (Bookari et al., 2016a). A recent Australian study surveyed pregnant women regarding exercise advice acquired from their antenatal care providers. Out of the 131 participants, 53% reported receiving some form of exercise with the frequency of exercise discussed among 34% of participants, while exercise duration was discussed only among 39% of the participants. Nonetheless, physical activity counselling during pregnancy has been found to be effective and feasible (Hayman et al., 2020). In a Finnish study, exercise interventions were delivered to 132 pregnant women by their antenatal care providers. Four face-to-face sessions, in addition to written materials describing benefits of exercise, goal setting, and tracking physical activity between clinical visits, were provided. The researchers found that the number of moderate-intensity physical activity days was 43% higher in those who received exercise counselling from their antenatal care provider, compared to pregnant women who were not counselled (Aittasalo et al., 2008).

Health professionals are often seen as trustworthy sources of information and during pregnancy women are more likely to attend multiple clinical appointments, making it an ideal time for behaviour change. Nevertheless, a US study examined health-care provider knowledge, beliefs, and practices regarding exercise during pregnancy using a cross-sectional 31-question survey. Overall, 99% of the respondents believed that exercise during pregnancy was beneficial. Despite this, a significant number of health-care providers claimed to be unfamiliar with the 1994 American College of Obstetricians and Gynaecologists guidelines for exercise during pregnancy (Bauer et al., 2010).

From an international perspective, various recommendations exist outlining the benefits and precautions associated with exercise during pregnancy (Aittasalo et al., 2008; Bauer et al., 2010). Australian physical activity guidelines advocate pregnant women to exercise on most days, aiming for 150–300 min of moderate-intensity physical activity each week (The Royal Australian and New Zealand College of Obstetricians and Gynaecologists RANZCOG, 2016). Despite well-documented health benefits of exercise, 60–80% of pregnant women do not participate in recommended amounts with commonly reported barriers including discomfort, fatigue, uncertainty about guidelines, and how to exercise safely (Evenson et al., 2009; The Royal Australian and New Zealand College of Obstetricians and Gynaecologists RANZCOG, 2016). Ultimately, the health of today’s children underpins the wellbeing of future populations thus health education should start before and during pregnancy (De Jersey et al., 2018). With increasing evidence suggesting pregnant women are not following healthy lifestyle recommendations, research should aim to examine possible reasons for this (Arrish et al., 2014, 2016; Bookari et al., 2016b; Christenson et al., 2018; De Jersey et al., 2013; Lucas et al., 2014; Weir et al., 2010). Therefore, the aim of this study was to explore the knowledge, beliefs, and practices of both pregnant women and health professionals in relation to physical activity and weight management in pregnancy.

## 2. Methods

Interpretative phenomenological analysis (IPA) is theoretically ensconced in both critical realism and the social cognition paradigm (Smith, 1996). The aim of IPA is to explore how participants make sense of their lived experiences and acknowledge how individuals may experience the “same” environment differently, producing subjective results (Draper & Swift, 2011; Fiske & Taylor, 1991). IPA emphasizes that the research is dynamic in nature involving a two-stage hermeneutic process as, first, the participants make sense of their world and, second, the researcher tries to interpret this in order to make sense of the participants’ world view (Draper & Swift, 2011).

Design

A semi-structured interview guide was developed by the research team based upon a review of the published literature (Arrish et al., 2016, 2017a; Bookari et al., 2016b; Cannon et al., 2020; De Jersey et al., 2013; Kraschnewski et al., 2014). Questions for both pregnant women and health professionals were similar and included “*How much physical activity do you believe is safe for a woman to undertake during pregnancy”? (Please also include your thoughts around frequency, intensity, duration and type)* and *“What is your understanding of the relationship between physical activity in pregnancy and birth outcomes such as having a normal vaginal delivery verses a c-section”?* In addition, health professionals were asked *“Do you refer to current best practice guidelines for physical activity in pregnancy”?* The majority of questions were kept deliberately open, providing cues for participants to talk with a minimum of interruption and without judgement. A purposive sampling technique was employed as this facilitated the selection of participants whose experiences were central to the study's aim. According to Smith et al., an adequate sample size is one that sufficiently answers the research question, the goal being to obtain cases rich in information (Smith, 1996). As such, 11 participants were deemed to be an adequate number, particularly as saturation was achieved following the eleventh interview Bracketing was not considered necessary as Heideggerian phenomenology espouses that it is impossible to negate human experiences related to the phenomenon under study resulting in personal awareness being intrinsic to phenomenological research. As such, reflexivity was practiced by maintaining an open dialogue, among the entire study team, throughout the research process.^23–24.^

Setting

A university campus, situated in a metropolitan region of southeastern Australia, was chosen as the main site for recruitment, with study information circulated via the institution’s intranet and via online social media platforms. This campus includes two general practitioner (GP) clinics and a school of midwifery as part of the faculty of health. It is common for pregnant women in Australia to select a shared care arrangement between their GP and local public hospital. Alternatively, some women choose to receive their antenatal care from a private practicing Obstetrician or Midwife.

Participants

Eligibility criteria for pregnant women included those aged ≥18 years, at least 12-week gestation, experiencing an uncomplicated pregnancy, and accessing antenatal care through the local area health service. Eligibility criteria for health professionals included those currently working in antenatal care (including midwives, obstetricians, and general practitioners). Individuals who agreed to participate were provided with information pamphlets outlining details of the study. Signed consent was given by each participant prior to interviews being conducted.

Data Collection

The two interviewers, BC and SC, attended training on interview techniques to ensure a standardized process was followed. This was conducted by the lead, CRKA, who is an experienced qualitative researcher. Basic demographic data was collected including age and country of birth. Women provided self-reported height and weight, which allowed calculation of BMI using the World Health Organization’s classification system (World Health Organisation, Regional Office for Europe, 2017). All health professionals were registered in their field with years of service kept confidential to ensure participants were not identifiable. Audio-recorded individual interviews were undertaken between October and November 2018 and took up to 60 min with the average length being 30 min for both women and health professionals. Interviews were conducted in each participant’s setting of choice to promote both comfort and privacy. The interviews were transcribed verbatim by BC and SC within 24 h of being held. Following the eleventh interview, data collected to date were scrutinized by the entire research team. Saturation was declared as no new information was obtained from the final interview with either the pregnant woman or health professional.

### Data Analysis

Data were analysed using the protocol for IPA published by Smith and Shinebourne (Smith & Shinebourne, 2012). Repeated reading of interview data was undertaken by the lead researchers, with each transcript allocated a pseudonym to ensure anonymity. Every line within each transcript was numbered to enable location of specific information during all stages of the analytical process. Data were coded and clustered into groups. When this had been repeated for each transcript, the resulting preliminary themes were examined to pinpoint recurrent patterns across the entire dataset. A final set of superordinate themes were decided following a round table discussion with the entire research team. Finally, corresponding quotes were assigned to each of these themes to demonstrate findings. Quotes were given a unique identifier comprising first characters to link the participants and second to indicate the line(s) from which the extract in question was taken. Thus, quotes termed “HP” referred to health professionals and “P” for pregnant women, with “HP1, L42” indicating the quote was taken from health professional 1 and begins on line 42 of the transcript. Differences in coding were discussed within the research team by presenting arguments for interpretation. Agreement was always reached following this process (Smith & Shinebourne, 2012). All data were stored on a password protected computer file, with only the study researchers having access to this information.

Ethics

The project was approved by the relevant Human Research Ethics Committee and adhered to the Declaration of Helsinki for experiments involving humans.

## 3. Results

Participating pregnant women (*n* = 6) had an average age of 27.5 years and a gestational age of 26 weeks, with further demographic data detailed in Table I. Participating health professionals consisted of midwives (*n* = 4) and one obstetrician (*n* = 1) with further participant demographic data outlined in Table II. Results have been detailed under three main themes, with quotes to demonstrate findings.

**Table I.** Demographic details of pregnant women interviewed.

Demographic variables	Category of respondents	Result
Pre-pregnancy BMI class	18.5–24.9 kg/m^2^ (Healthy)	2
	24.9–29.9 kg/m^2^ (Overweight) 30+kg/m (Australian Institute of Health and Welfare, 2017) (Obese)	3 1
Country of birth	Australia	5
	Bangladesh	1
Gestational age	12–25 weeks	3
	26–40 weeks	3
Location of antenatal care	Public	5
	Private	1

BMI—body mass index.

**Table II.** Demographic details of health professionals interviewed.

Participant ID	Gender	Profession	Time working as health professional in antenatal care (years)
01	Male	Obstetrician Gynaecologist	>20
02	Female	Registered Midwife	<20
03	Female	Registered Midwife and Gynaecology Nurse	>20
04	Female	Registered Midwife	<20
05	Female	Registered Midwife	<20

### 3.1 Women rely on multiple sources of pregnancy-related healthy lifestyle information

The world-wide web has become a very popular source of health information for pregnant women. With the internet being readily accessible, women felt: *“It’s one of the easiest places … . to find stuff quickly”* (P1, L155). Clinicians expressed the belief that a significant number of women use the internet as a primary source of health information such as physical activity during pregnancy: “*A lot of women google things of course, and a lot of the time they want to find out if what they have googled is actually right” (HP01)*. This raises the important issue of resource credibility. Of concern is that pregnant women may either choose not to or are not given the opportunity to discuss what they learn about physical activity from the internet with their antenatal health-care provider.

Family, friends, and health professionals outside of the antenatal clinic also influence healthy lifestyle behaviours such as physical activity with a common belief that pregnancy is a time “to rest and take it easy”. Frustration regarding this “cotton wool” attitude was expressed by one health professional: *“Even (some) GPs tell them to stop doing exercise when they come and see us … that to me smells like culture it doesn’t smell like evidence and unfortunately culture eats evidence for breakfast … if she’s told by her GP and her mum and her sister … that’s really hard for a midwife to unpack” (*HP05, L131). Likewise, women reported receiving contradictory information regarding whether they should continue or cease exercise during pregnancy. One participant made the point that “old school” advice still abounds: *“My mum’s way of thinking is that all women should probably be at home in bed not doing a lot … .”* (P4, L167). Conversely, another woman believed*: “ …… . the view of exercise during pregnancy has changed so much in our generation from maybe walking around the block with some hand weights to really doing everything that (was done) before”* (P3, L173). Unfortunately, pregnant women must often deal with advice from their social networks that differs from mainstream biomedical sources. Such contradictory views may lead to feelings of confusion and anxiety resulting in the possibility that some women don’t end up following best practice guidance even if their intention was to do so.

Maintaining a good level of exercise during the antenatal period was acknowledged by health professionals as a strong indicator of a woman’s ability to cope with labour or to birth naturally: *“I think it’s important that pregnant women … . maintain a degree of physical fitness … or they might end up with a caesarean section”* (HP01, L17). Health professionals reported being largely supportive about the benefits of exercise during pregnancy recognizing that both maternal and neonatal outcomes may be significantly influenced by a woman’s fitness levels.

Despite most health professionals claiming to encourage women to maintain their pre-pregnancy exercise levels, such advice was usually brief and lacked robust content: *“What we know, what we’ve learnt ourselves (is what we provide regarding healthy lifestyle behaviours). … so, we probably talk very generally.”* (HP03, L45). Conversely, pregnant women reported that lifestyle-related discussions were rarely initiated by their antenatal care providers with one participant making the point: *“I have noticed that unless you ask your midwife or doctor a question … they don’t tell you so without that information you would have to come up with the questions on your own, so there would be certain things that you do that you didn’t realize were wrong until someone told you.”* (P2, L79). Essentially women felt that health professionals were not as forthcoming with lifestyle-related advice as they would have liked. This seemed to be in stark comparison to the belief held by the health professionals that they were.

### 3.2 Discussions around healthy lifestyle behaviours are low priority and often inconsistent

Written information was often used as a substitute for lifestyle-related discussions, with one woman making the comment: *“She printed out on her computer a forty-page (pregnancy-specific nutrition and physical activity) brochure … she was so adamant in giving it to me, so I assumed it was important.”* (P2, L201).

The amount of content that needs to be covered in an initial antenatal visit is considerable. As such, health professionals find it challenging to prioritize conversations around healthy lifestyle behaviours, such as regular physical activity during pregnancy: *“I’m not aware of too many midwives going into healthy lifestyle advice in great detail because unfortunately there’s so many other things we’ve got to discuss.”* (HP03, L81).

Most women reported not being weighed during pregnancy holding the assumption that if they: “ … … *had gone in underweight or overweight it might have been a different story … ”* (P3, L192). Other women pointed out that as they were not routinely weighed maintaining (and tracking) a healthy GWG was a real challenge. One way to negate this was to undertake what was felt to be an appropriate amount of physical activity by: *“listening to (my) body and knowing not to push too hard”* (P1, L76).

Clinicians’ knowledge of appropriate GWG ranged broadly from “9 kg to 20 kg” with some believing that it was not *“ …… their place to bring (the topic) up with women”* (HP03, L70). Others expressed the opinion that for women with a high pre-pregnancy BMI *“ …… it’s too late to do anything now so don’t sort of dwell on it”* (HP03, L70).

Health professionals claimed that pre-pregnancy BMI determines what lifestyle advice, if any, is provided. For those women who present to the antenatal clinic within a healthy BMI, clinicians routinely tell them: “ … … *not to stress about it, you know if someone puts on 5 kg during a pregnancy, but the baby is well grown, I* don’t *see that as an issue, I* don’t *see that as a problem …* (however*) the women* themselves *tend to see it as a* problem.” (HP01, L90). Concern was expressed that lifestyle-related advice such as the amount and type of physical activity that is recommended was not individually tailored and generally impersonal with one pregnant woman stating: *“I think it was more like just a tick of the box kind of thing on the first set of questions … it was never anything specific*.” (P4, L79).

Health professionals acknowledged that pregnancy-specific physical activity best practice guidelines would exist (and be accessible through the antenatal clinic), however, considerable gaps in knowledge regarding these was demonstrated: *“I mean …… I don’t actually know about the guidelines”* (HP02, L101) and *“to be completely honest with you, I wouldn’t know what or where they were … I tend not to read guidelines.”* (HP01, L120). Such comments highlight the importance of multidisciplinary advocacy in antenatal care and that midwives and obstetricians may benefit from additional support from allied health colleagues such as Exercise Physiologists.

### 3.3 Lifestyle-related topics perceived as sensitive make some conversations and actions difficult

Health professionals acknowledged weight as a sensitive topic to speak openly about, with one clinician expressing concern about how women feel if such discussions occur: *“We have to be very careful and also what we say to women around their weight because some can get defensive and its personal … .”* (HP04, L104). Others recognized that: “*staff find it difficult to approach (issues such as weight) with the women … some midwives are probably better than it than others, some feel confident, some don’t …… ”* (HP03, L70). Stories of negative experiences were recalled by health professionals of pregnant women who had attended specialist bariatric services: *“Those women will (then) come to a general antenatal clinic and say ‘I don’t want to go back there because I don’t want to be reminded every time I come how fat I am, and it just makes me feel awful.”* (HP01, L73). Such experiences left some women feeling stigmatized which in turn soured what otherwise should have been a happy time for them.

Women expressed concern that health professionals working in routine antenatal care did not always bring up the topic of weight or how to manage it through behaviours, such as physical activity. As a result, many were fearful of complications that may accompany weight fluctuation during the antenatal period: *“putting on too much or too little weight during pregnancy could even be an indicator of some issue or could cause other issues” (P5, L21)*. However, others felt that pregnancy was an opportunity to relax healthy lifestyle behaviours*: “ …… an excuse not to exercise and eat for two and all that kind of stuff” (P4, L21)*. On the flip side, health professionals expressed concern that some pregnant women become anxious and even obsessive when the topic of weight is mentioned claiming that women often make comments such as: *“’Oh I’ve put on sooooo much weight’ or ‘how much weight should I have put on?’”* (HP02, L67). It was acknowledged that long-standing societal taboos make it difficult for some women to actively maintain a high level of exercise during pregnancy with one health professional expressing sentiments previously voiced: *“I think there’s a feeling in culture that women need to wrap themselves in cotton wool … when they’re pregnant.”* (HP05, L 128). Ultimately, the general feeling was that women were left to their own devices regarding how to manage their gestational weight gain as described by the following participant: *“I informed myself through books …… to up my knowledge on the benefits of different kinds of exercise”* (P4, L159).

## 4. Discussion

This study explored the knowledge, practices, and beliefs of a group of pregnant women and health professionals, regarding physical activity and weight management behaviours during the antenatal period. Health professionals acknowledged the role of exercise during pregnancy and how it may assist with labour and prevention of a caesarean section. However, health professionals in our study failed to identify other well-documented benefits of physical activity such as the prevention and/or management of GDM plus the positive influence on mood or other physiological advantages including posture and muscle tone. Some women in our study acknowledged the beneficial effects of physical activity during pregnancy but claimed they were left primarily to their own devices regarding how much to undertake and at what intensity. A recent US retrospective cohort study assessed physical activity frequency prior to and during pregnancy in addition to health professional provider advice. The authors found that few women met American College of Obstetrics and Gynaecology guidelines for physical activity. Nearly one-third of women did not receive advice about physical activity during the antenatal period and, of concern, was that obese women were no more likely to receive advice than their normal weight counterparts (Santo et al., 2017).

The findings of our study reinforced existing literature that physical activity discussions between health professionals and women are of low priority compared to other pregnancy-related topics (World Health Organisation, Regional Office for Europe, 2017). Even though best practice exercise guidelines exist for pregnancy in Australia, the health professionals interviewed in our study were unable to single these out (The Royal Australian and New Zealand College of Obstetricians and Gynaecologists RANZCOG, 2016). Similarly, survey research from the UK found only 2% of midwives were able to correctly identify physical activity guidelines during pregnancy (Hopkinson et al., 2018). Published literature has also reported that while pregnant women have been provided with lifestyle-related brochures outlining topics such as healthy eating and recommended levels of physical activity, in the absence of a verbal discussion with a health professional, these often remain unread (Szwajcer et al., 2009).

Health professionals in our study acknowledged that conversations around weight may be avoided due to the sensitive nature of the topic. Previous research has referred to obesity as a “conversation stopper” with health professionals acknowledging being extra cautious not to embarrass, upset, or negatively impact the emotional well-being of women regarding the topic of weight (Chang et al., 2013; Johnson et al., 2013). In an Australian qualitative study, midwives reported weight-related discussions difficult to initiate especially with obese women (Willcox et al., 2012). Furthermore, time constraints, poor knowledge base, and the need to cover a myriad of other information during antenatal clinics were acknowledged by obstetricians and midwives in our study as major barriers to conversations around physical activity and lifestyle-related topics. Similar results have been published previously (Chang et al., 2013).

“Woman-centredness” recognizes a woman’s individual circumstances highlighting her physical, emotional, psychosocial, spiritual, and cultural needs, as documented in “The National Competency Standards for the Midwife”. With the “woman-centred” approach being pivotal to antenatal care, findings from our study juxtaposed this belief by identifying a lack of individualized and structured lifestyle-related pregnancy advice available to women. Some health professionals displayed a sense of “giving up” regarding the provision of such advice. This view is supported by previous findings where antenatal care clinicians perceived nutrition and exercise counselling to be ineffective and unlikely to support lifestyle-related behaviour changes (Knight-Agarwal et al., 2014). With the internet, a valuable resource for pregnant women, antenatal care facilities are in a prime position to support access to credible, evidence-based weight management, and exercise information, via a multitude of different platforms (Bookari et al., 2017; Furness et al., 2011; Huberty et al., 2013).

A literature review reported that the Australian Accreditation Standards for Nursing and Midwifery courses provide no content on lifestyle-related behaviours, such as physical activity and nutrition during pregnancy (Arrish et al., 2014). Similarly, in the Royal Australian and New Zealand College of Obstetricians and Gynaecologists (RANZCOG) integrated Training Programme, there is no specific module for physical activity, diet, or how to support women to undertake lifestyle-related behaviour change during pregnancy (RANZCOG, 2015). A 2018 systematic review examined the relationship between prenatal exercise, GWG, and postpartum weight retention. Eighty-four (*n* = 21 530) randomized controlled trials (RCTs) showed that exercise-only interventions decreased total GWG (*n* = 5819; −0.9 kg, 95% CI − 1.23 to −0.57 kg, I (Australian Institute of Health and Welfare, 2017) = 52%) and postpartum weight retention (*n* = 420; −0.92 kg, 95% CI − 1.84 to 0.00 kg, I (Australian Institute of Health and Welfare, 2017) = 0%) plus reduced the odds of excessive GWG (*n* = 3519; OR 0.68, 95% CI 0.57 to 0.80, I (Australian Institute of Health and Welfare, 2017) = 12%) compared with no exercise (Arrish et al., 2014). Given the prevalence of overweight and obesity in our society, optimal weight management before, during and after pregnancy is an important area of clinical practice. It has been well established that regular physical activity is one way to achieve this (Aittasalo et al., 2008). The presence of dedicated exercise physiologists in antenatal care may help to alleviate the many barriers faced by both pregnant women and health professionals, including obstetricians and midwives, in the giving and receiving of physical activity advice (Bauer et al., 2010). Consequently, a multidisciplinary approach should be considered as the “gold standard” in the application of “woman-centred care”.

As far as we are aware, this study is among the first to use an IPA framework to explore the knowledge, beliefs, and practices of Australian antenatal healthcare professionals and pregnant women in relation to physical activity and weight management. It is important to recognize that this study was small, and participants were not asked to identify their race or describe their social circumstances in detail. The location of the study is known to over-represent educated, middle class, English-speaking women. Traditionally, IPA studies are conducted with relatively small groups, with the aim being to find a *reasonably* homogeneous sample. However, we acknowledge that in future IPA investigations of this kind it would be useful to recruit women with similar BMIs (Smith, 1996) We also acknowledge that a clinician’s approach to discussing gestational weight and physical activity may be dependent on a woman’s BMI. Likewise, this is something we could explore in more detail if the sample of women interviewed (either underweight or healthy weight or overweight) were even more homogenous than the sample presented within our research. In addition, the recruitment strategy may be potentially biased to people interested in physical activity. Therefore, the views expressed here may not be representative of other health professionals and pregnant women in Australia. As the findings emerged through an interpretative lens, doubts may be created about the experimental claims that arose. However, co-researchers discussed codes and themes in relation to the phenomena in question, which according to Smith (1996) ensures the final account produced is a credible one (Smith, 1996). It is important to emphasize that the aim of this study was to explore the knowledge, beliefs, and practices of the target groups in relation to pregnancy-related lifestyle topics and was not intended to be an evaluation of current antenatal practice. We also acknowledge the importance of nutrition in weight management (Teede et al., 2022). However, there is evidence that dietary information is more readily available to women during pregnancy compared to physical activity information (Cannon et al., 2020). We therefore wanted to explore some of the potential reasons for this. Future research would benefit from delving into both lifestyle issues within a pregnant population.

## 5. Conclusion

The findings from this qualitative investigation suggest that gaps exist in the knowledge of antenatal healthcare professionals and pregnant women regarding key lifestyle-related topics, such as gestational weight management and physical activity. Both groups in our study confirmed that conversations around these topics are not happening unless specific questions are asked and, even then, answers are not always forthcoming, or evidence based. In addition, a lack of time and confidence, as well as the sensitive nature of some issues such as weight, has been cited as important barriers to the sharing of lifestyle-related information. Significant evidence exists regarding the efficacy of multidisciplinary interventions for the promotion of healthy lifestyle behaviours in a range of patient groups, particularly cardiac rehabilitation (Jackson et al., 2018). However, more research is needed in relation to acceptability and cost-effectiveness of routine exercise physiology inclusion within Australian antenatal care settings. Nevertheless, midwives and obstetricians may find benefits in collaborating with exercise physiologists regarding optimal physical activity advice and behaviour change techniques for pregnancy. The themes generated by this study may form the foundation for future investigations and, in turn, contribute to clinical policy and best practice guidelines regarding healthy weight management and physical activity advice in Australian antenatal care settings.
